# Real-world effectiveness of *Yindan Jiedu* granules-based treatment on patients infected with the SARS-CoV-2 Omicron variants BA.2 combined with high-risk factors: A cohort study

**DOI:** 10.3389/fphar.2022.978979

**Published:** 2022-08-16

**Authors:** Ying Feng, Yao Liu, Long Liu, Yao Liu, Yuyong Jiang, Yixin Hou, Yang Zhou, Rui Song, Xiaoyou Chen, Xianbo Wang

**Affiliations:** ^1^ Department of Integrative Medicine, Beijing Ditan Hospital, Capital Medical University, Beijing, China; ^2^ Department of Infectious Diseases, Beijing Ditan Hospital, Capital Medical University, Beijing, China; ^3^ Beijing Ditan Hospital, Capital Medical University, Beijing, China

**Keywords:** *Yindan jiedu* granules, Paxlovid, SARS-CoV-2 Omicron variant, clinical research, high-risk factors, COVID-19

## Abstract

**Background:** Our previous studies have shown that *Yindan Jiedu* granules (YDJDG) can effectively treat coronavirus disease 2019 (COVID-19); however, the high infectivity and the immune escape potential of the Omicron variant BA.2 make it more difficult to control, and patients with high-risk factors prone to progress rapidly.

**Purpose:** To evaluate YDJDG’s efficacy in treating patients with the Omicron variant BA.2 with high-risk factors and compared it with that of Paxlovid.

**Methods:** A total of 257 patients who fulfilled the inclusion criteria were allocated to the YDJDG (115 cases), Paxlovid (115 cases), and control (27 cases) groups. A Cox regression model was used to analyze the independent factors affecting the shedding time of nucleic acid in 14 days. Propensity score matching (PSM) was used to match the characteristics of individuals in the three groups, while the Kaplan-Meier method was used to compare the shedding proportion of nucleic acids.

**Results:** Cox analysis showed that the vaccine booster (*p* = 0.006), YDJDG treatment (*p* = 0.020), and Paxlovid treatment (*p* < 0.0001) were independent predictors of nucleic acid shedding at 14 days. The median recovery time was 11.49 days in the YDJDG group, 10.21 days in the Paxlovid group, and 13.93 days in the control group. After PSM (3:1), the results showed that the nucleic acid shedding time of the YDJDG group (*n* = 53) was 2.47 days shorter than that of the control group (*n* = 21) (*p* = 0.0076), while the Paxlovid group (*n* = 44) had a 4.34 days shorter than that of the control group (*n* = 17) (*p* < 0.0001). After PSM (1:1), YDJDG and Paxlovid (76 pairs) were also analyzed. In the YDJDG group, nucleic acid shedding time was 1.43 days longer than that observed in the Paxlovid group (*p* = 0.020). At 10 and 14 days, the Paxlovid group showed a significant difference in the nucleic acid shedding proportion compared with the control group (*p* = 0.036, *p* = 0.0015). A significant difference was also observed between the YDJDG and control groups (*p* = 0.040) at 14 days.

**Conclusion:** As a safe and convenient oral drug, YDJDG can be used as an alternative to antiviral therapy for such patients.

## Introduction

Since December 2019, the coronavirus disease 2019 (COVID-19), caused by SARS-CoV-2, has spread worldwide. As of May 31, 2022, the number of confirmed infections exceeded 529,347,278, with 6,288,930 deaths (https://coronavirus.jhu.edu/map.html). The Omicron (B.1.1.529) variant quickly replaced the Delta and became the leading variant worldwide(Paton et al., 2022). To date, the Omicron variant BA.2 is the most infectious variant, and its infectivity is about 1.5 and 4.2 times that of the BA.1 strain and Delta, respectively. Moreover, due to frequent mutations in the receptor binding domain (RBD) in the receptor binding motif (RBM) of the S protein, the immune escape potential of the BA.2 was 30% higher than that of the BA.1 and 17 times higher than that of the Delta variant(Chen and Wei, 2022). Although it is believed that the clinical symptoms of the Omicron variant are less severe than those of the Delta variant (Wolter et al., 2022), patients with high-risk factors may still progress rapidly and become life-threatening. In general, the prevention and control of COVID-19 become more difficult.

Currently, the primary treatment methods approved by the FDA for patients with COVID-19 are monoclonal antibodies (mAbs) and oral antiviral drugs (https://www.covid19 treatmentguidelines.nih.gov/). Unfortunately, similar to vaccines, mAbs are vulnerable to viral mutations. The Omicron variant optimizes RBD mutations of the RBM, strengthening its binding force with the host angiotensin converting enzyme-2 (ACE-2) and enhancing its infectivity, making it difficult to neutralize with most antibodies produced by vaccinations or previous infections (Chen and Wei, 2022). Therefore, the effect of most mAbs against RBM is seriously impaired by the Omicron subvariant, and their clinical treatment effect on patients infected with the BA.2 is limited.

Paxlovid (nirmatrelvir/ritonavir) is one of the most representative antiviral drugs (McDonald and Lee, 2022). Nirmatrelvir inhibits the activity of SARS-CoV-2 major protease (MPRO) and restricts virus replication, and the addition of ritonavir, a strong CYP3A inhibitor, can increase the blood concentration of nirmatrelvir and enhance its therapeutic effect. Therefore, when Paxlovid is combined with highly dependent drugs on CYP3A metabolic clearance or CYP3A strong inducers, drug interactions can occur easily (Heskin et al., 2022). However, many patients with high-risk factors require various drugs to control their disease. Once taken Paxlovid, they need to suspend or replace the current drugs and even require more monitoring of the blood drug concentration, such as digoxin. It is difficult to adjust the current treatment in the 5-days window period, which may affect the control of basic diseases. In addition, Paxlovid is not recommended for patients with severe renal insufficiency (EGFR <30 ml/min) and severe liver insufficiency (Child–Pugh grade C). Therefore, there are various restrictions on the use of Paxlovid.

*Yindan Jiedu* granules (YDJDG) is a Chinese botanical drugs produced by combining the *Maxing Shigan* and *Qingwen Baidu* decoctions, prescribed as a newly applied Chinese herbal formula at the Beijing Ditan Hospital. Since January 2020, the YDJDG has been used to treat patients with COVID-19 at the Beijing Ditan Hospital. Our previous clinical study confirmed that, compared with lopinavir-ritonavir treatment or routine treatment, YDJDG could hasten the recovery period in patients with COVID-19 by shortening the SARS-CoV-2 nucleic acid shedding time, the mean duration of fever, and exudative pulmonary lesions (Liu et al., 2020a; Feng et al., 2022). We also revealed that one of the mechanisms by which YDJDG can shorten the course of COVID-19 and delay its progression is the inhibition of inflammation by targeting the NF-κB pathway (Feng et al., 2022).

However, the efficacy of YDJDG on the Omicron variant BA.2, the primary epidemic strain in the world, has not been studied. Moreover, there are few studies on the therapeutic effect of Chinese traditional medicine (TCM) in patients with COVID-19 with high-risk factors. Furthermore, the efficacy of YDJDG was not compared with that of antiviral drugs, such as Paxlovid, leading to the fact that the advantages and effects of YDJDG were not fully reflected.

## Patients and methods

### Patients

**FIGURE1:**
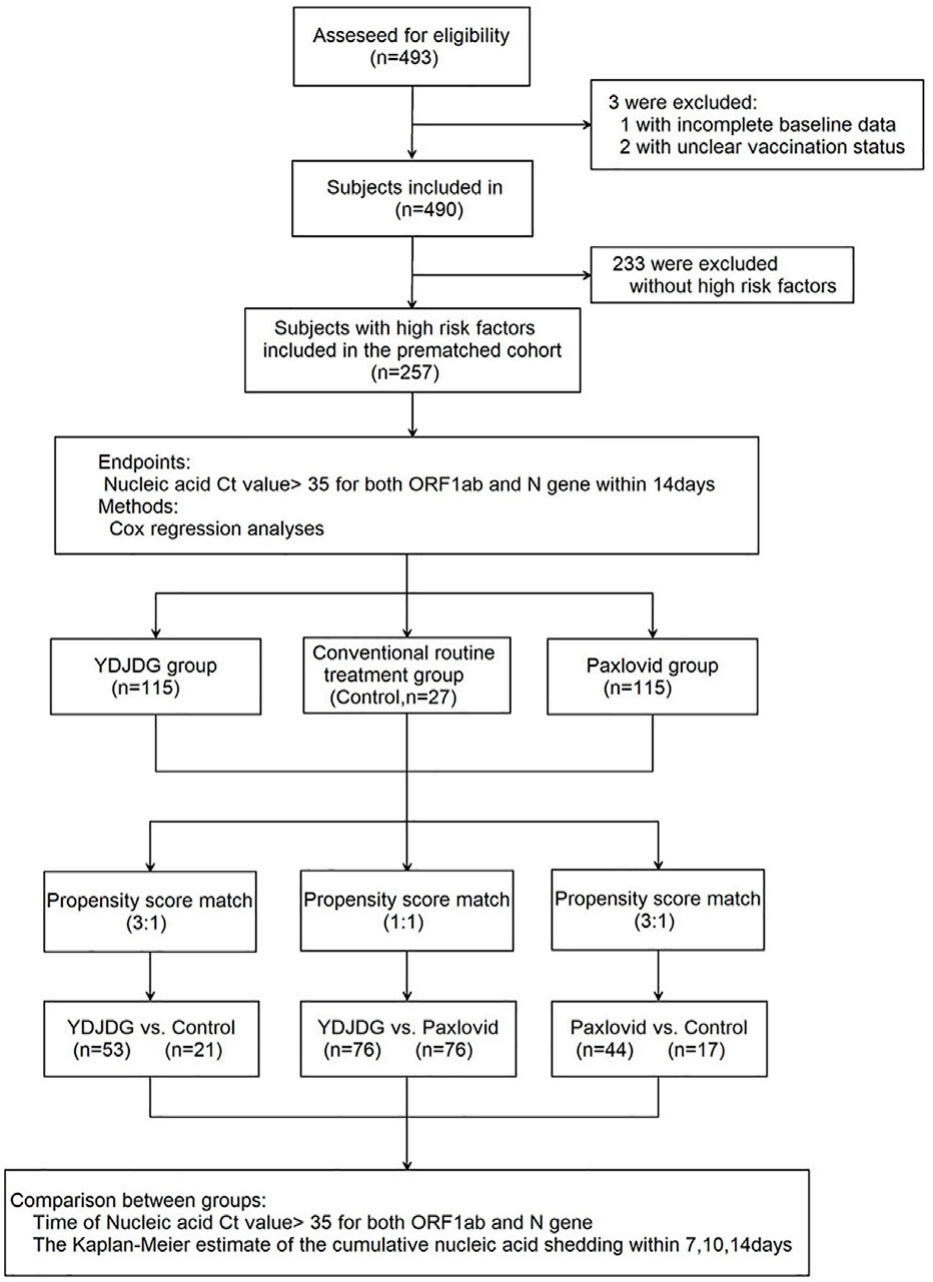
Flow chart of the clinical research. A total of 493 participants were screened for eligibility; 257 patients were included in the research. The Cox regression model was used to analyze the independent factors affecting the nucleic acid shedding time after a follow-up of 14 days. Propensity score matching (PSM) was used to match the characteristics of individuals in the three groups. The nucleic acid shedding time and proportion of nucleic acid shedding at 14 days were compared separately. COVID-19, coronavirus disease 2019; YDJDG, *Yindan Jiedu* granules.

A total of 493 patients infected with the SARS-CoV-2 Omicron variant BA.2 hospitalized at the Beijing Ditan Hospital between April 22, 2022, and May 24, 2022, were recruited. The inclusion criteria and the clinical classifications are as follows: confirmed cases of COVID-19, which was diagnosed and classificated based on the new coronavirus pneumonia diagnosis and treatment plan (trial version 9) developed by the National Health Committee of the People’s Republic of China (http://www.nhc.gov.cn/). The exclusion criteria were described in a paper previously published by our research group. (Liu et al., 2020a; Liu et al., 2020b). All positive RT-PCR tests were screened using an Omicron-specific RT-PCR test, verified by whole genome sequencing. Finally, 257 patients with more than one high-risk factors were enrolled in the study. Among them, 20 cases were classificated as asymptomatic type, 194 cases were mild type, 43 cases were moderate type, and there were no severe and critical type patients. The high-risk factors are defined as the following, according to the Diagnosis and Treatment Protocol for Coronavirus Pneumonia (Trial version 9): ① old age (≥60 years old); ② obese or overweight (BMI ≥30 kg/m^2^); ③ current heavy smokers; ④ hypertension or cardiovascular disease (including congenital heart disease); ⑤ diabetes; ⑥ chronic lung diseases (such as chronic obstructive pulmonary disease, asthma, interstitial lung disease, cystic fibrosis, and pulmonary hypertension); ⑦ chronic kidney disease; ⑧ chronic liver diseases, including cirrhosis, nonalcoholic fatty liver, hepatitis B or hepatitis C virus hepatitis and alcoholic liver disease; ⑨ active cancer; and ⑩ metabolic syndrome. Among them, 115 received YDJDG therapy, 115 received Paxlovid treatment, and 27 received routine treatment (Figure 1). This study was approved by the Institutional Research Ethics Committee of the Beijing Ditan Hospital, Capital Medical University (Beijing, China). All the patients provided signed informed consent.

### Drug preparation and treatment

The YDJDG was approved by the Beijing Medical Products Administration (China) (No. Z20200012000) and was produced according to the Pharmacopeia of the People’s Republic of China. It is composed of *Ephedra sinica* Stapf [*Ephedraceae; Ephedrae Herba*], *Lonicera japonica* Thunb [*Caprifoliaceae; Lonicerae Japonicae Flos*], *Morus alba* L. [*Moraceae; Mori Cortex*], *Lepidium apetalum* Willd [*Cruciferae; Lepidii Semen*], *Scutellaria baicalensis* Georgi [*Lamiaceae; Scutellariae Radix*], *Actaea heracleifolia* (Kom.) J.Compton [*Ranunculaceae; Cimicifugae Rhizoma*], *Scutellaria baicalensis* Georgi [*Scrophulariaceae; Scutellariae Radix*], *Paeonia suffruticosa* Andrews [*Ranunculaceae; Moutan Cortex*], *Rehmannia glutinosa* (Gaertn.) DC.[ *Scrophulariaceae; Rehmanniae Radix*], *Atractylodes macrocephala* Koidz [*Asteraceae; Atractylodis Macrocephalae Rhizoma*], *Gypsum* [*Sulfates; Gypsum Fibrosum*] (Beijing Medicinal Materials Company, Beijing, China). The production method has been reported in our previous study (Liu et al., 2020a). Routine treatment generally consists of supportive treatments, such as oxygen and symptomatic therapies, at the discretion of attending clinicians. The dose of YDJDG was 12 g or 24 g, which was administered orally three times per day. The Paxlovid treatment was co-packaged as nirmatrelvir (two 150 mg tablets) with ritonavir (one 100 mg tablet); the three tablets were taken together twice daily for five days. The antiviral and routine treatment plans were based on the Diagnosis and Treatment Protocol for Coronavirus Pneumonia (Trial version 9) and the notice on adjusting the indications for the COVID-19 drug pf-07321332/ritonavir (Paxlovid) tablets issued by the National Health Commission.

### Observation outcome

The main outcome was nucleic acid shedding time, defined as the time from the first positive test to the first day of nucleic acid cycle threshold (Ct) value >35 for both ORF1ab and N genes.

### Propensity score matching

To reduce bias in the analysis, a 1:1 or 1:3 PSM was used. The YDJDG group was matched with the Paxlovid group at a 1:1 ratio according to the propensity scores generated using a caliper width of 0.1. The sample size of the control group was small; therefore, the control group was matched with the YDJDG or Paxlovid groups at a 1:3 ratio according to the generated propensity scores using a caliper width of 0.1. The variables for this procedure were median age, sex, median days from onset of illness to admission time, number of high-risk factors, disease severity, number of symptoms, vaccination, and Ct value of the initial SARS-CoV-2 RT-PCR tests. The baseline characteristics of the patients with COVID-19 before and after PSM are shown in Tables 2–4.

### Statistical analysis

Statistical analyses were conducted using the GraphPad Prism (version 8.00, CA, United States), SPSS (version 22.0, IBM, NY, United States), and R (version 4.1.2, R Foundation for Statistical Computing, Vienna, Austria) software packages (Survive and MatchIt). Variables are expressed as numbers (%). Age, days from illness onset to admission time, and continuous variables are expressed as median (interquartile interval). The cutoff values for continuous variables were calculated based on mean values. The differences between the two groups were determined using the *t*-test for continuous variables with normal distribution, the Mann–Whitney *U* test for continuous variables that did not have a normal distribution, and the Chi-square test for count data. Univariate and multivariate Cox regression analyses were performed to identify independent factors affecting the shedding time of nucleic acids. The Kaplan-Meier method was used to compare the shedding proportion of nucleic acids at different time points. The Mantel–Cox method was used to compare the log-rank values between more than two groups. Statistical significance was set at *p*-value < 0.05.

## Results

### Nuclear acid shedding time in all 257 participants

**FIGURE 2:**
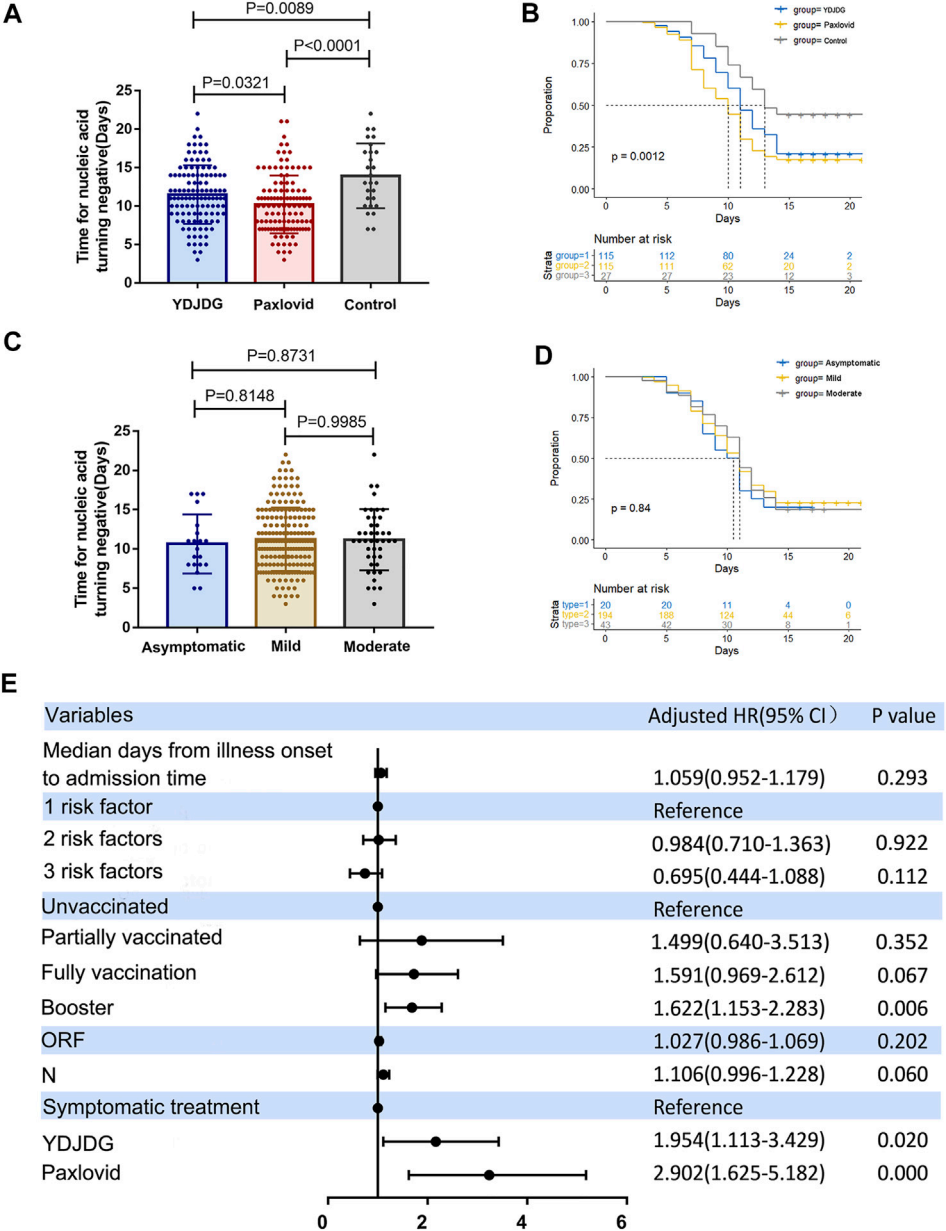
Analysis of 257 participants enrolled in the study. **(A)** The median SARS-CoV-2 nucleic acid shedding time was 10.21 days in the Paxlovid group, 11.49 days in the YDJDG group, and 13.93 days in the control group. **(B)** Significant differences were observed among the three groups in the SARS-CoV-2 nucleic acid shedding rate at 14 days (χ2 = 13.45, *p* = 0.0012). There was no statistical difference of nucleic acid shedding time **(C)** and shedding rate at 14 days **(D)** among patients with different disease types (*p* > 0.05). **(E)** Cox regression analyses for nucleic acid shedding proportion in patients with COVID-19 after a follow-up of 14 days. Vaccine booster, YDJDG therapy, and Paxlovid therapy are significant factors affecting nucleic acid shedding (*p* < 0.05). COVID-19, coronavirus disease-2019; YDJDG, *Yindan Jiedu* granules.

A total of 493 patients were screened for eligibility, and 257 were included in this study. The median SARS-CoV-2 nucleic acid shedding time of the participants was 10.21 days in the Paxlovid group, 11.49 days in the YDJDG group, and 13.93 days in control group (Figure 2A). There was a significant difference in the SARS-CoV-2 nucleic acid shedding rate at 14 days among the three groups (χ2 = 13.45, *p* = 0.0012) (Figure 2B). Meanwhile, there was no statistical difference of nucleic acid shedding time and shedding rate at 14 days among patients with different disease types (Figures 2C,D).

Based on the findings of the univariate and multivariate Cox regression analyses, booster vaccine (HR = 1.622, 95% CI: 1.153–2.283, *p* = 0.006), YDJDG treatment (HR = 1.954, 95% CI: 1.113–3.429, *p* = 0.020), and Paxlovid treatment (HR = 2.902, 95% CI: 1.625–5.182, *p* < 0.0001) were independent prognostic factors for SARS-CoV-2 nucleic acid shedding after a follow-up of 14 days. (Table 1; Figure 2E).

**TABLE 1:** Univariate and multivariate Cox regression analyses for SARS-CoV-2 nucleic acid shedding after a follow-up of 14 days.

Variables	Univariate analysis			Multivariate analysis		
	β	HR (95%CI)	*p*-value	β	HR (95%CI)	*p*-value
Age	−0.004	0.996(0.987–1.005)	0.401	—	—	—
Sex	0.079	1.082(0.818–1.431)	0.581	—	—	—
Median days from illness onset to admission time	0.076	1.079(0.978–1.191)	0.131	0.057	1.059(0.952–1.179)	0.293
No. of High risk factors	—	—	0.133	—	—	0.264
1	0	Ref	—	0	Ref	—
2	0.168	1.183(0.874–1.601)	0.276	−0.016	0.984(0.710–1.363)	0.922
3	−0.295	0.744(0.481–1.151)	0.184	−0.364	0.695(0.444–1.088)	0.112
Disease severity	—	—	0.806	—	—	—
Asymptomatic	0	Ref	—	—	—	—
Mild	−0.126	0.881(0.488–1.593)	0.676	—	—	—
Moderate	−0.169	0.845(0.504–1.415)	0.521	—	—	—
No.of symptoms	—	—	0.943	—	—	—
0	0	Ref	—	—	—	—
1	0.037	1.038(0.669–1.61)	0.868	—	—	—
2	−.034	0.966(0.606–1.541)	0.886	—	—	—
3	−0.07	0.932(0.6–1.447)	0.754	—	—	—
Vaccination	—	—	0.051	—	—	0.046
Unvaccinated	0	Ref	—	0	Ref	—	
Partially vaccinated	0.498	1.646(0.702–3.857)	0.252	0.405	1.499(0.64–3.513)	0.352
Full vaccination	0.508	1.662(1.013–2.726)	0.044	0.464	1.591(0.969–2.612)	0.067
Booster	0.459	1.583(1.128–2.221)	0.008	0.484	1.622(1.153–2.283)	0.006
Initial SARS-CoV-2 RT-PCR tests						
ORF	0.026	1.027(0.987–1.068)	0.189	0.026	1.027(0.986–1.069)	0.202
N	0.029	1.03(0.991–1.07)	0.135	0.101	1.106(0.996–1.228)	0.060
Treatment	—	—	0.004	—	—	0.002
Symptomatic treatment	0	Ref	—	0	Ref	—
YDJDG	0.574	1.776(1.028–3.067)	0.039	0.670	1.954(1.113–3.429)	0.020
Paxlovid	0.865	2.374(1.375–4.099)	0.002	1.066	2.902(1.625–5.182)	0.000

### Clinical characteristics of participants

A total of 74 participants were selected after a 3:1 PSM between the YDJDG and control groups, 53 from the YDJDG group and 21 from the control group (Table 2). After another 3:1 PSM between the Paxlovid and control groups, 61 participants were selected, 44 from the Paxlovid group and 17 from the control group (Table 4). After a 1:1 PSM between the YDJDG and Paxlovid groups, 152 participants were selected, 76 each from the YDJDG and Paxlovid groups (Table 3). There was almost no significant difference between the two groups in median age, sex, median days from onset of illness to admission time, number of high-risk factors, disease severity, number of symptoms, vaccination, and Ct value of initial SARS-CoV-2 RT-PCR tests, respectively (Tables 2–4).

**TABLE 2:** Baseline characteristics of COVID-19 patients before and after propensity score matching between YDJDG and Control group.

	Before propensity score matching			After propensity score matching(3:1)		
Variables	YDJDG	Control	*p* Value	YDJDG	Control	*p* Value
	(*n* = 115)	(*n* = 27)		(*n* = 53)	(*n* = 21)	
Median Age (range)	54(40–63)	53(45–62)	0.882^a^	52(35.5–58.5)	49(40–60)	0.703^a^
Sex, Male, n (%)	65(56.5)	12(44.4)	0.260^c^	27(50.9)	11(52.4)	0.913^c^
Median days from illness onset to admission time P50 (P25-75)	1(1–2)	1(0–2)	0.430^b^	1(1–2)	1(1–2)	0.545^b^
No. of High risk factors, n (%)						
1	68(59.1)	22(81.5)	0.030^c^	45(84.9)	16(76.2)	0.374^c^
2	29(25.2)	5(18.5)	0.463^c^	4(7.5)	5(23.8)	0.125^c^
3	18(15.7)	0(0)	0.024^c^	4(7.5)	0(0)	0.572^c^
Disease severity, n (%)						
Asymptomatic	10(8.7)	6(22.2)	0.045^c^	4(7.5)	2(9.5)	1.000^c^
Mild	90(78.3)	20(74.1)	0.639^c^	43(81.1)	18(85.7)	0.641^c^
Moderate	15(13)	1(3.7)	0.297^c^	6(11.3)	1(4.8)	0.668^c^
No. of symptoms, n (%)						
0	16(13.9)	7(25.9)	0.127^c^	7(13.2)	3(14.3)	1.000^c^
1	34(29.6)	8(29.6)	0.995^c^	16(30.2)	8(38.1)	0.512^c^
2	32(27.8)	4(14.8)	0.249^c^	15(28.3)	4(19)	0.599^c^
3	33(28.7)	8(29.6)	0.923^c^	15(28.3)	6(28.6)	0.982^c^
Vaccination, n (%)						
Unvaccinated	24(20.9)	8(29.6)	0.327^c^	16(30.2)	5(23.8)	0.583^c^
Partially vaccinated	3(2.6)	1(3.7)	1.000^c^	1(1.9)	1(4.8)	1.000^c^
Full vaccination	8(7)	3(11.1)	0.744^c^	3(5.7)	3(14.3)	0.451^c^
Booster	80(69.6)	15(55.6)	0.164^c^	33(62.3)	12(57.1)	0.684^c^
Initial SARS-CoV-2 RT-PCR tests						
ORF, P50(P25-75)	23.8(21.2–26.2)	22.2(20.4–24.3)	0.063^b^	24.1(22.8–26.2)	22.5(21.5–25.1)	0.218^b^
N, P50(P25-75)	20.7(17.9–23.3)	19.9(17.9–22.5)	0.303^b^	21.0(20.1–23.7)	20.0(19.0–22.6)	0.319^b^

a *p* values comparing YDJDG and Control group are from *t* test,

b Mann-Whitney *U* test, or

c *χ2* test.

COVID-19, 2019 coronavirus disease. YDJDG, Yindan Jiedu granule.Symptoms including: fever, dry cough, fatigue, anosmia of smell and taste, stuffy nose, runny nose, sore throat, conjunctivitis, myalgia and diarrhea.

**TABLE 3:** Baseline characteristics of COVID-19 patients before and after propensity score matching between YDJDG and Paxlovid group.

	Before propensity score matching			After propensity score matching(1:1)		
Variables	YDJDG	Paxlovid	*p* Value	YDJDG	Paxlovid	*p* Value
	(*n* = 115)	(*n* = 115)		(n = 76)	(*n* = 76)	
Median Age (range)	54(40–63)	65(57–70)	0.001^a^	58.5(52.25–65)	62(52–68)	0.237^a^
Sex, Male, n (%)	65(56.5)	69(60)	0.595^c^	44(57.9)	48(63.2)	0.510^c^
Median days from illness onset to admission time P50 (P25-75)	1(1–2)	1(1–2)	0.885^b^	1(1–2)	1(1–2)	0.836^b)^
No. of High risk factors, n (%)						
1	68(59.1)	44(38.3)	0.002^c^	40(52.6)	30(39.5)	0.104^c^
2	29(25.2)	52(45.2)	0.001^c^	22(28.9)	34(44.7)	0.044^c^
3	18(15.7)	19(16.5)	0.858^c^	14(18.4)	12(15.8)	0.667^c^
Disease severity, n (%)						
Asymptomatic	10(8.7)	4(3.5)	0.168^c^	5(6.6)	3(3.9)	0.716^c^
Mild	90(78.3)	84(73)	0.357^c^	60(78.9)	58(76.3)	0.697^c^
Moderate	15(13)	27(23.5)	0.041^c^	11(14.5)	15(19.7)	0.389^c^
No.of symptoms, n (%)						
0	16(13.9)	14(12.2)	0.695^c^	9(11.8)	8(10.5)	0.797^c^
1	34(29.6)	40(34.8)	0.397^c^	22(28.9)	32(42.1)	0.090^c^
2	32(27.8)	22(19.1)	0.120^c^	24(31.6)	18(23.7)	0.276^c^
3	33(28.7)	39(33.9)	0.394^c^	21(27.6)	18(23.7)	0.577^c^
Vaccination, n (%)						
Unvaccinated	24(20.9)	35(30.4)	0.097^c^	15(19.7)	15(19.7)	1.000^c^
Partially vaccinated	3(2.6)	3(2.6)	1.000^c^	2(2.6)	1(1.3)	1.000^c^
Full vaccination	8(7)	16(13.9)	0.084^c^	4(5.3)	11(14.5)	0.103^c^
Booster	80(69.6)	61(53)	0.010^c^	55(72.4)	49(64.5)	0.295^c^
Initial SARS-CoV-2 RT-PCR tests						
ORF, P50(P25-75)	23.8(21.2–26.2)	22.0(20.6–24.8)	0.023^b^	23.4(20.7–25.9)	22.7(20.9–25.3)	0.715^b^
N, P50(P25-75)	20.7(17.9–23.3)	19.1(17.4–22.0)	0.009^b^	20.3(17.6–23.1)	19.8(17.7–22.2)	0.963^b^

a *p* values comparing YDJD group and Paxlovid group are from *t* test,

b Mann-Whitney *U* test, or

c *χ2* test.

COVID-19, 2019 coronavirus disease. YDJDG, Yindan Jiedu granule.Symptoms including: fever, dry cough, fatigue, anosmia of smell and taste, stuffy nose, runny nose, sore throat, conjunctivitis, myalgia and diarrhea.

**TABLE 4:** Baseline characteristics of COVID-19 patients before and after propensity score matching between Paxlovid and control group.

	Before propensity score matching			After propensity score matching(3:1)		
Variables	Paxlovid	Control	*p* Value	Paxlovid	Control	*p* Value
	(*n* = 115)	(*n* = 27)		(*n* = 44)	(*n* = 17)	
Median Age (range)	65(57–70)	53(45–62)	0.001^a^	62(52.3–67.8)	53(47.5–61)	0.190^a^
Sex, Male, n (%)	69(60)	12(44.4)	0.144^c^	24(54.5)	10(58.8)	0.768^c^
Median days from illness onset to admission time P50 (P25-75)	1(1–2)	1(0–2)	0.530^b^	1(1–2)	1(0.5–1.5)	0.571^b)^
No. of High risk factors, n (%)						
1	44(38.3)	22(81.5)	0.000^c^	28(63.6)	12(70.6)	0.608^c^
2	52(45.2)	5(18.5)	0.011^c^	13(29.5)	5(29.4)	0.992^c^
3	19(16.5)	0(0)	0.024^c^	3(6.8)	0(0)	0.553^c^
Disease severity, n (%)						
Asymptomatic	4(3.5)	6(22.2)	0.003^c)^	3(6.8)	1(5.9)	1.000^c^
Mild	84(73)	20(74.1)	0.913^c)^	37(84.1)	15(88.2)	0.995^c^
Moderate	27(23.5)	1(3.7)	0.040^c)^	4(9.1)	1(5.9)	1.000^c^
No. of symptoms, n (%)						
0	14(12.2)	7(25.9)	0.070^c^	6(13.6)	2(11.8)	1.000^c^
1	40(34.8)	8(29.6)	0.610^c^	21(47.7)	7(41.2)	0.645^c^
2	22(19.1)	4(14.8)	0.806^c^	6(13.6)	4(23.5)	0.582^c^
3	39(33.9)	8(29.6)	0.670^c^	11(25)	4(23.5)	1.000^c^
Vaccination, n (%)						
Unvaccinated	35(30.4)	8(29.6)	0.935^c^	15(34.1)	6(35.3)	0.929^c^
Partially vaccinated	3(2.6)	1(3.7)	1.000^c^	0(0)	1(5.9)	0.279^c^
Full vaccination	16(13.9)	3(11.1)	0.944^c^	8(18.2)	1(5.9)	0.417^c^
Booster	61(53)	15(55.6)	0.814^c^	21(47.7)	9(52.9)	0.715^c^
Initial SARS-CoV-2 RT-PCR tests						
ORF, P50(P25-75)	22.0(20.6–24.8)	22.2(20.4–24.3)	0.573^b^	21.9(19.5–24.9)	22.5(21.5–25.1)	0.938^b^
N, P50(P25-75)	19.1(17.4–22.0)	19.9(17.9–22.5)	0.567^b^	19.5(17.2–22.2)	20.0(19.0–22.6)	0.541^b^

a *p* values comparing Paxlovid group and Control group are from *t* test,

b Mann-Whitney *U* test, or

c χ^2^ test.

COVID-19, 2019 coronavirus disease.Symptoms including: fever, dry cough, fatigue, anosmia of smell and taste, stuffy nose, runny nose, sore throat, conjunctivitis, myalgia and diarrhea.

### Nucleic acid shedding time between the three groups

**FIGURE 3:**
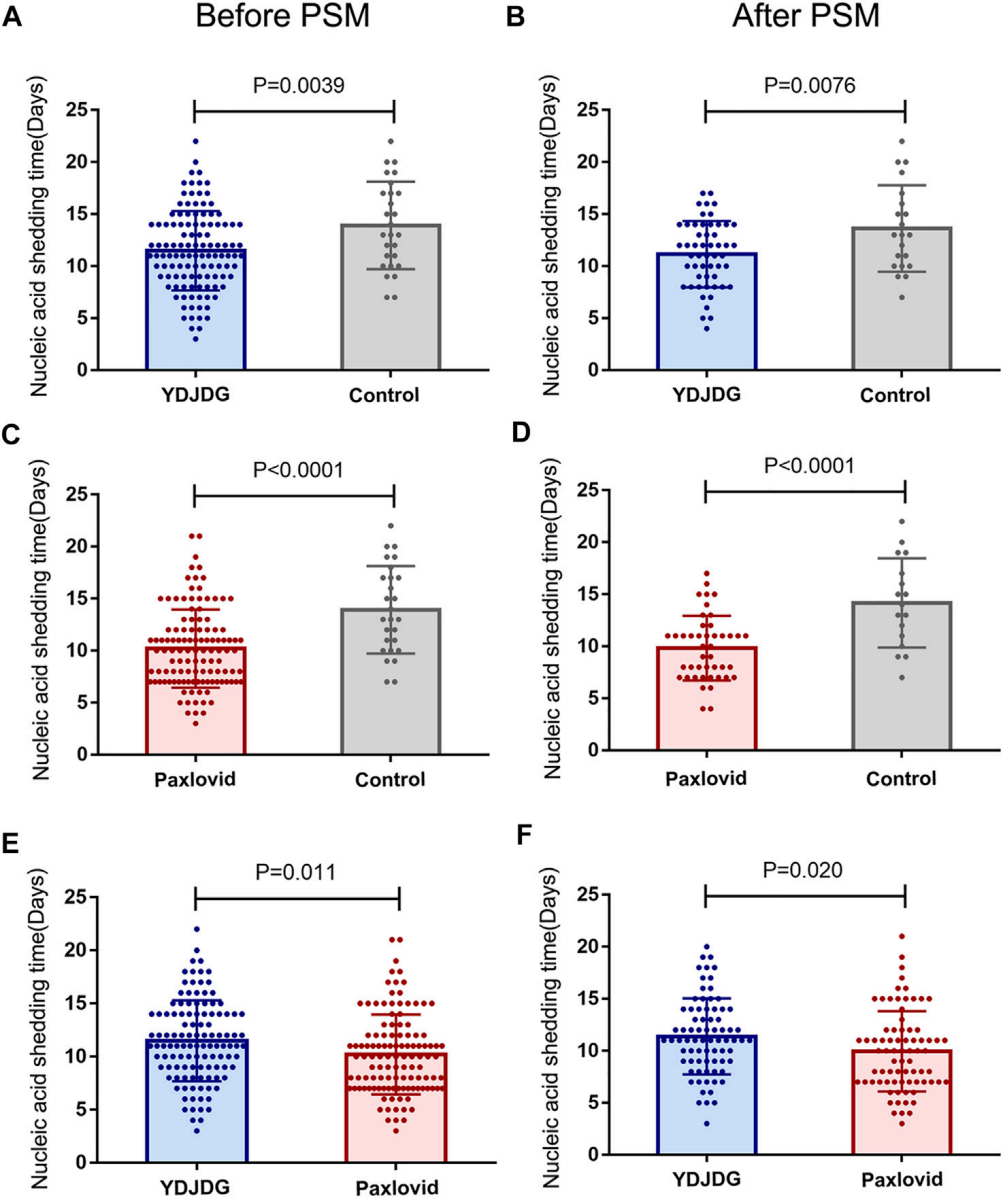
The nucleic acid shedding time before and after PSM. Comparison of the YDJDG and the control group before **(A)** and after **(B)** PSM. Comparison of the Paxlovid and the control group before **(C)** and after **(D)** PSM. Comparison of the YDJDG and the Paxlovid group before **(E)** and after **(F)** PSM. YDJDG, *Yindan Jiedu* granules; PSM, propensity score matching.

Compared with the control group, the YDJDG group (median: 11.49 *vs.* 13.93 days, *p* = 0.0039) and the Paxlovid group (median: 10.21 *vs.* 13.93 days, *p* < 0.0001) had a significantly shorter time of negative conversion of viral nucleic acid, respectively (Figures 3A,C). After PSM, the time of negative conversion of viral nucleic acid in the YDJDG group (median: 11.15 *vs.* 13.62 days, *p* = 0.0076) and the Paxlovid group (median: 9.84 *vs.* 14.18 days, *p* < 0.0001) was still shorter than that in the control group (Figures 3B,D). The time to negative conversion of viral nucleic acid in the Paxlovid group was significantly shorter than that in the YDJDG group before PSM (median: 10.21 *vs.* 11.49 days, *p* = 0.011, Figure 3E) and after PSM (median: 9.96 *vs.* 11.39 days, *p* = 0.020, Figure 3F).

**FIGURE 4:**
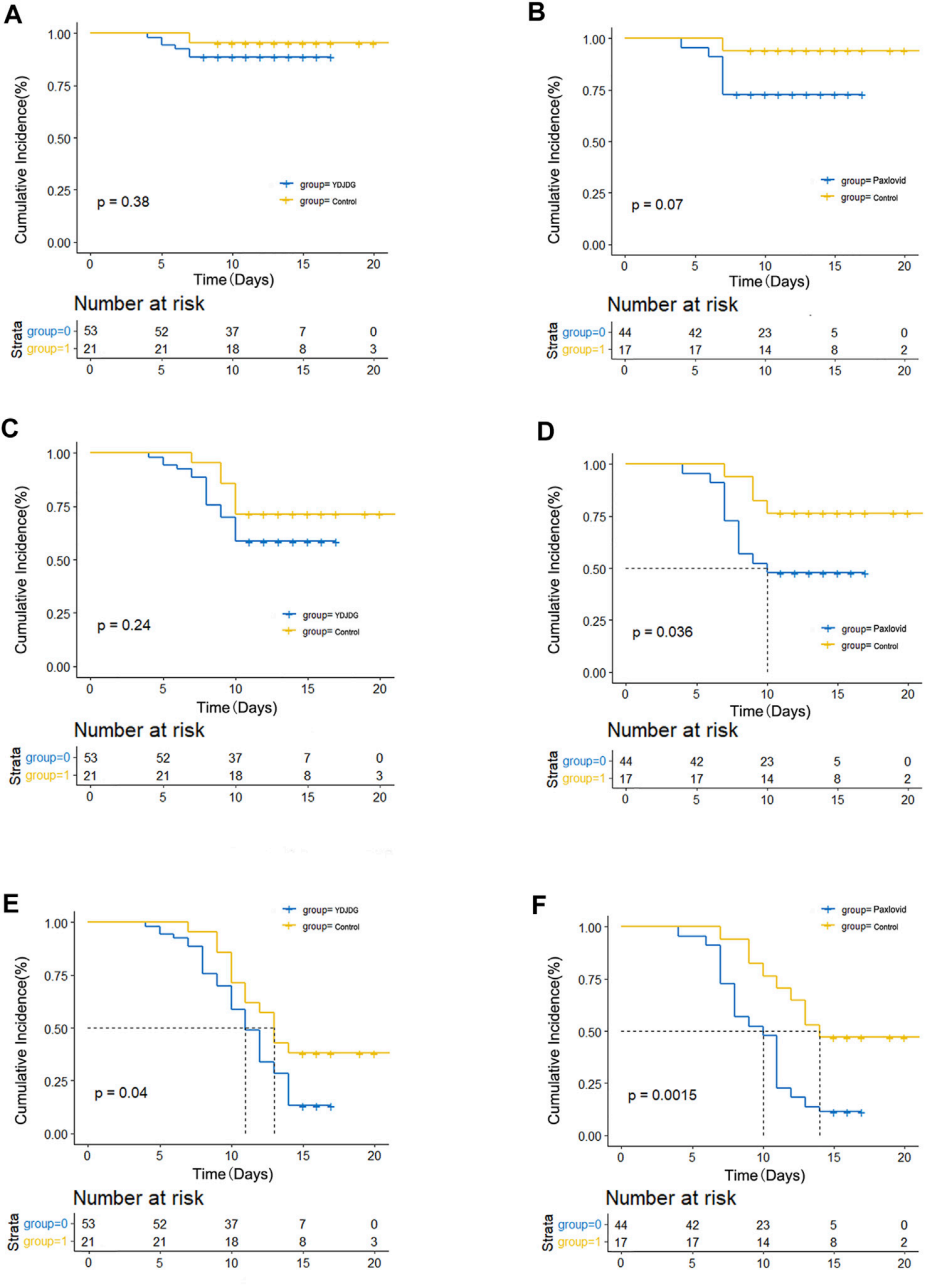
Kaplan–Meier estimates of proportion recovery. Proportion recovery estimates are shown in patients after PSM. The nucleic acid shedding proportion was compared between the YDJDG and the control group after a follow-up of 7 days **(A)**, 10 days **(C)**, and 14 days **(E)**. The nucleic acid shedding proportion was compared between the Paxlovid and the control group after a follow-up of 7 days **(B)**, 10 days **(D)**, and 14 days **(F)**. YDJDG, *Yindan Jiedu* granules; PSM, propensity score matching.

Furthermore, at 7, 10, and 14 days, the proportion of nucleic acid shedding was compared among the YDJDG, Paxlovid, and control groups (Figures 4A–F). The results revealed that at 10 and 14 days, the Paxlovid group showed a significant difference in proportion compared with the control group (*p* = 0.036, *p* = 0.0015, Figures 4D,F). The proportion of nucleic acid shedding time was also significant in the YDJDG group (*p* = 0.040) at 14 days (Figure 4E).

## Discussion

According to the whole genome sequencing results, all patients admitted to the Beijing Ditan Hospital from April 22, 2022, to May 24, 2022, had the SARS-CoV-2 Omicron variant BA.2. The total number of confirmed cases in this round of the epidemic has exceeded all previous rounds in Beijing, suggesting that the Omicron variant BA.2 has strong infectivity and greater difficulty in overall prevention and control.

Although the existing evidence suggests that the clinical symptoms caused by the Omicron variant and its subvariants are mild (Meo et al., 2021; Kim et al., 2022), patients with more than one high-risk factor are still prone to disease progression. Thus it is meaningful to focus on the treatment plan of such patients. According to the Diagnosis and Treatment Protocol for Coronavirus Pneumonia (Trial version 9), the main standard for discharge is a nucleic acid Ct value >35 two consecutive times, with an interval of 24 h. Therefore, it is of great significance to shorten the nucleic acid shedding time of patients to save more medical resources.

Our data analysis showed that the average time of nucleic acid shedding (13.93 days) in patients with more than one high-risk factor was significantly longer than the previously reported 11.13 days in patients taken no account of risk-factors (Shen et al., 2022). Although patients with high-risk factors prone to progress rapidly, there was no significant difference in the time of nucleic acid shedding with one or more high-risk factors and one or more symptoms, even the original nucleic acid load and disease classification. However, vaccination and oral medication (YDJDG or Paxlovid) were independent factors affecting the negative conversion of nucleic acids.

At present, the novel coronavirus vaccines that have been developed in clinics mainly include inactivated vaccines, genetic engineering vaccines (subunit vaccines, nanoparticle vaccines, viral vector vaccines), and nucleic acid vaccines (DNA vaccines, RNA vaccines) (McDonald et al., 2021). The effects of most of the monoclonal antibodies and recombinant vaccines against RBM of the S protein have been seriously impaired due to the multipoint mutation of the RBD of the S protein in the Omicron variant(Cameroni et al., 2021; VanBlargan et al., 2021). However, the vaccines inoculated in China are the main inactivated vaccines, which have been proved significantly reduced the risk of symptomatic COVID-19, and serious adverse events were rare in prespecified interim analysis of a randomized clinical trial (Al Kaabi et al., 2021). Although they need to be administered more than twice and the immunization cycle is long, it has the advantage of complete immunogenicity because immunogenicity is aimed at the whole virus. Our analysis results showed that vaccine booster (HR = 1.622, 95% CI: 1.153–2.283*, p* = 0.006) was an independent prognostic factor of nucleic acid shedding within 14 days, which fully confirmed the role of three doses of inactivated vaccine in fighting against the Omicron variant infection, consistent with previous reports (Zeng et al., 2022).

In the antiviral treatment for patients with high-risk factors after PSM, the median nucleic acid shedding time of patients in the Paxlovid group was 9.96 days, which was 1.43 days less than that in the YDJDG group (11.39 days). The average conversion time of patients in the YDJDG group was shorter by 2.47 days (median: 11.15 *vs.* 13.62 days, *p* = 0.0076) compared with the control group. Moreover, compared with Paxlovid, which has strict requirements for drugs involving the CYP3A metabolic pathway, while basic disease drugs does not need to be stopped when taking YDJDG, making YDJDG an important and safe choice for patients with various serious basic diseases and severe liver and kidney function damage(Liu et al., 2020a). In addition, our previous studies have confirmed that one of the main functions of YDJDG in treating COVID-19 is to reduce the body’s inflammatory response, which has a positive effect on inhibiting severe diseases. Furthermore, compared with the 5-days window period required by Paxlovid, YDJDG is simple to administer, has good safety, and is easy to obtain, making it a good choice for patients with COVID-19 in addition to small-molecule drugs.

## Conclusion

In patients infected with the SARS-CoV-2 Omicron variant combined with high-risk factors, YDJDG can significantly shorten nucleic acid shedding time. Compared with Paxlovid group, the gap of the median nucleic acid shedding time between the two groups is less than 2 days. Considering the characteristics of convenient administration, lower price and less drug interaction, YDJDG is a good choice for patients with COVID-19. However, our study had several limitations. First, this study was not randomized, controlled, and double-blinded; the number of samples in the control group was small; therefore, a 1:1 or 1:3 PSM was used to reduce baseline bias. In addition, because this study aimed to compare the nucleic acid shedding time, the serum inflammatory index of the groups was not compared. In the future, randomized, controlled, and double-blind trials with a large sample size will be carried out to confirm further the efficacy of YDJDG in treating COVID-19.

## Data Availability

The datasets presented in this article are not readily available because the raw data required to reproduce these findings cannot be shared at this time as the data also forms part of an ongoing study. Requests to access the datasets should be directed to wangxb@ccmu.edu.cn.
