# Supplementary figures and images for: Real-world effectiveness of Yindan Jiedu granules-based treatment on patients infected with the SARS-CoV-2 Omicron variants BA.2 combined with high-risk factors: A cohort study
Source: Front Pharmacol. 2022 Aug 16;13:978979. doi: 10.3389/fphar.2022.978979 (PMC9426238; doi:10.3389/fphar.2022.978979)

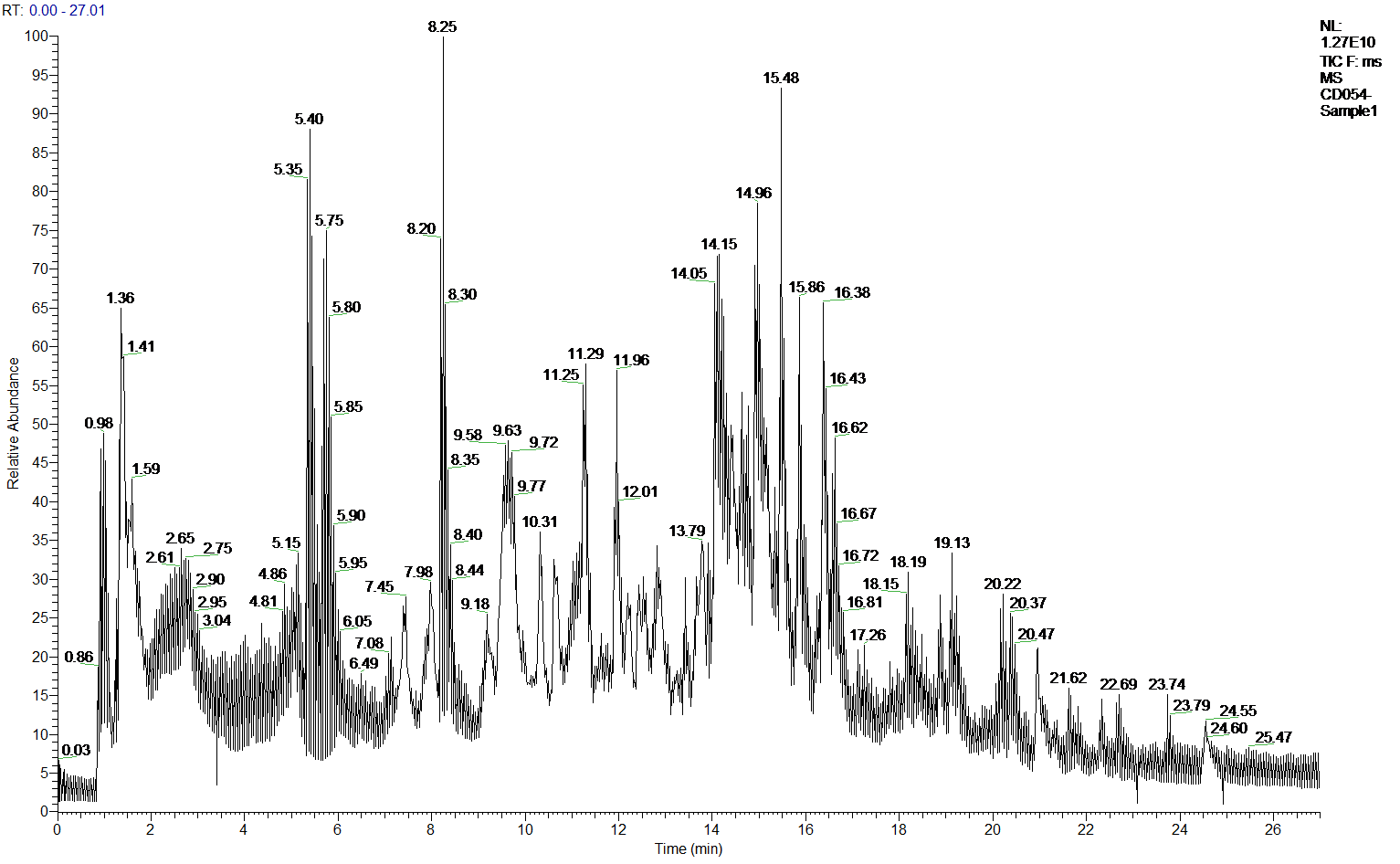

Supplement: Supplementary file 1 [file Image2.TIF]

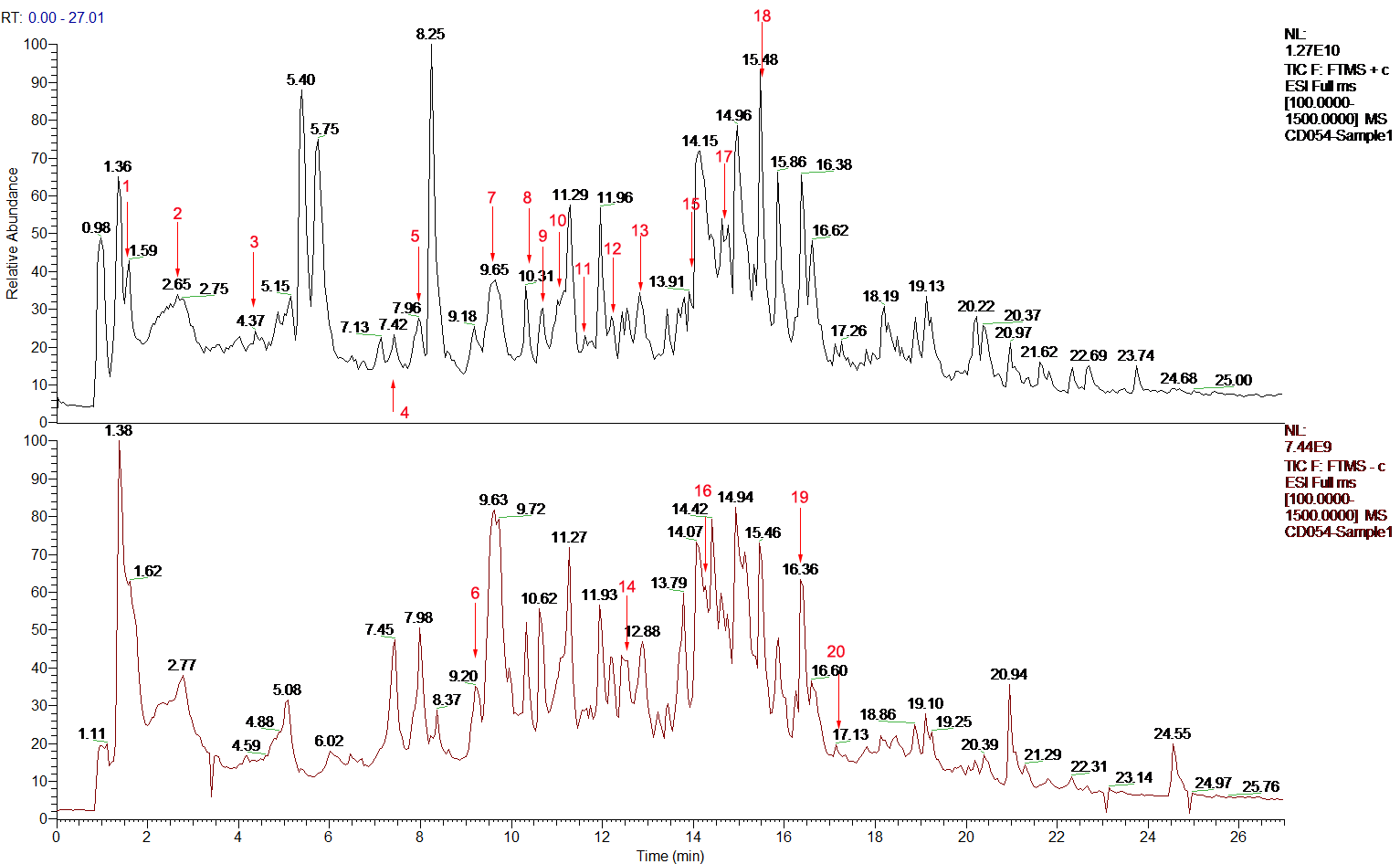

Supplement: Supplementary file 2 [file Image1.TIF]
